# Diversity and evolution of quorum-sensing systems in *Rhizobium*

**DOI:** 10.3389/fbinf.2026.1767204

**Published:** 2026-04-17

**Authors:** Ivana Blancas-Nava, Erick Cruz-Santiago, Gabriela Guerrero, Rosa-Maria Gutierrez-Rios, Miguel A. Cevallos

**Affiliations:** 1 Centro de Ciencias Genómicas, Programa de Genómica Evolutiva, Universidad Nacional Autónoma de México, Cuernavaca, Morelos, Mexico; 2 Instituto de Biotecnología, Departamento de Microbiología Molecular, Universidad Nacional Autónoma de México, Cuernavaca, Morelos, Mexico; 3 Centro de Ciencias Genómicas, Unidad de Análisis Bioinformático, Cuernavaca, Morelos, Mexico

**Keywords:** horizontal gene transfer, LuxI, LuxR, plasmids, Rhizobium

## Abstract

Quorum-sensing (QS) systems based on acyl-homoserine lactones (AHLs) regulate gene expression in response to cell density in many bacteria, including *Rhizobium*. These systems, typically composed of LuxI-like synthases and LuxR-like regulators, control processes such as plasmid conjugation, biofilm formation, and plant interactions. However, their evolutionary dynamics and genomic distribution in *Rhizobium* remain poorly understood. We analyzed 142 complete *Rhizobium* genomes using comparative genomics, phylogenetic reconstruction, and genomic context analysis. LuxI/LuxR homologs were identified based on sequence similarity and Pfam domain architecture, and their genomic contexts were examined. Phylogenetic relationships and coevolution between LuxI/LuxR pairs were assessed using cophylogenetic approaches. QS systems showed a highly heterogeneous distribution across *Rhizobium* genomes: some strains lacked canonical systems, whereas others encoded one or multiple systems in chromosomes and/or plasmids. Chromosomal QS systems were associated with multiple distinct genomic contexts, supporting at least seven independent acquisition events. In contrast, plasmid-encoded systems exhibited substantially greater diversity in both sequence and genomic organization. Phylogenetic and comparative analyses revealed dynamic gains and losses of QS systems, variable coevolution among LuxI/LuxR pairs, and evidence of partner recruitment. Notably, plasmids appear to act as major reservoirs of QS systems and likely sources of their transfer to chromosomes. These findings indicate that QS systems in *Rhizobium* evolve through a combination of horizontal gene transfer, genomic rearrangement, and differential retention across replicons. The higher diversity and mobility of plasmid-encoded systems highlight their central role in shaping QS evolution and functional innovation. Overall, this study provides a comprehensive framework for understanding the diversification and evolutionary trajectories of QS systems in complex multipartite bacterial genomes.

## Introduction

Quorum sensing (QS) is a widespread cell-to-cell communication mechanism that allows bacterial populations to synchronize their gene expression in a density-dependent manner. Moreover, many quorum-sensing systems (QSSs) enable bacteria to detect the presence of other bacterial species and even eukaryotic organisms, allowing them to respond accordingly (Miller and Bassler, 2001; Hughes and Sperandio, 2008).

QSS activity depends on the synthesis and secretion of a signaling molecule called an autoinducer and its detection by specific transcription factors. The most common signaling molecules in many Pseudomonadota (Proteobacteria) are acyl-homoserine lactones (AHLs) (Kumar et al., 2022). These molecules comprise a conserved homoserine lactone ring linked to an acyl chain, which can vary in length (from C_4_ to C_20_), and they have varying oxidation states at the C_3_ position and degrees of unsaturation (Fuqua et al., 2001). These autoinducers are produced by AHL synthases (LuxI-like) and are detected by LuxR-like transcription factors, proteins that are homologous to those involved in the regulation of bioluminescence in the marine bacterium *Aliivibrio fischeri* (Verma and Miyashiro, 2013). LuxI-like AHL synthases are cytoplasmic enzymes that use S-adenosylmethionine (SAM) and an acyl-ACP as substrates for the production of autoinducers (Moré et al., 1996). In turn, LuxR-like transcription factors comprise proteins with two functional domains: a helix-turn-helix DNA-binding domain in the carboxy-terminal region, which recognizes DNA sequences (lux-like boxes) within QS-regulated promoters, and a second domain at the amino-terminal region that binds the autoinducer signaling molecule (Fuqua et al., 2001; Choi and Greenberg, 1992). The crystal structures of several LuxR-like proteins bound to their cognate AHL have been determined, revealing the critical amino acid residues that recognize the AHL (He et al., 2017; Vannini et al., 2002; Bottomley et al., 2007; Zhang et al., 2002).

In the canonical configuration, the genes encoding LuxI-like and LuxR-like proteins are located next to each other in different arrangements: they are encoded in tandem on the same DNA strand or on opposite strands in either a tail-to-tail or head-to-head configuration (Gelencsér et al., 2012; Cai and Zhang, 2024). However, bacterial genomes are being increasingly reported to contain *lux*I-like and *lux*R-like gene pairs separated by a few genes, which may play a relevant role in the QS regulatory circuitry (Cai and Zhang, 2024). In general, the proteins encoded by QS genes in the canonical configuration work together: the LuxR-like transcriptional regulator responds to the autoinducer produced by the cognate LuxI-like protein (Gelencsér et al., 2012; Cai and Zhang, 2024), suggesting that these proteins have coevolved (Wellington Miranda et al., 2021).

In many bacterial genomes, genes encoding LuxR-like transcriptional regulators are located several kilobases from each other or without LuxI-like genes. These types of LuxR-like proteins are known as orphans or solos. These transcriptional regulators play crucial roles in cell physiology by expanding the repertoire of endogenous and exogenous AHLs that the bacteria harboring them can detect (Patankar and González, 2009; Subramoni and Venturi, 2009; Xu, 2020; Venturi et al., 2015). In many organisms, the autoinducer binding domain has evolved and has lost the amino acids critical for autoinducer binding; as a result, these organisms no longer respond to the presence of AHLs but they are capable to respond to substances other than AHLs, and in some cases, they play important roles in interkingdom communication (i.e., plant–microbe interactions) (Zhang et al., 2007).

*Rhizobium* is an alpha-proteobacterial genus comprising species that form tight associations with plants. Most of its members make endosymbiotic associations with the roots of compatible legume plants, inducing the formation of nitrogen fixation nodules (Yang et al., 2022). However, other genus members, such as *R. rhizogenes*, *R. radiobacter* and *R. tumorigenes* are plant pathogens (Flores-Félix et al., 2020) (Kuzmanović et al., 2018).

A notable characteristic of many *Rhizobium* species is their ability to synthesize a wide range of AHLs. This ability is due largely to the presence of multiple QSSs encoded within a single *Rhizobium* genome. Moreover, frequently LuxI-like synthases can produce more than one type of AHLs. Another key feature is the frequent presence of numerous *luxR* solo genes. Furthermore*, luxI-*like and *luxR-*like genes in *Rhizobium* are encountered in diverse genetic contexts (Calatrava-Morales et al., 2018; Sanchez-Contreras et al., 2007; González and Marketon, 2003; Wisniewski-Dyé and Downie, 2002).

QS is involved in a wide range of *Rhizobium* functions that can be broadly classified into three categories: a) Physiological functions, such as biofilm formation, exopolysaccharide synthesis regulation, bacterial motility, and growth arrest (Dixit et al., 2017; Acosta-Jurado et al., 2021; Wilkinson et al., 2002); b) functions involved in plasmid physiology, such as plasmid conjugation and copy number control (Dixit et al., 2017; Cervantes et al., 2019; Ding and Hynes, 2009; Danino et al., 2003); and c) functions related to their symbiotic/pathogenic relationship with plants, for example, disruption of QSSs can reduce nodulation efficiency or provoke alterations of adhesion to plant cell surfaces (Miao et al., 2018; Zheng et al., 2015; Soto et al., 2006). However, importantly, some QS responses can be classified into more than one of these categories.

The study of the mechanisms involved in the evolution of QSS in *Rhizobium* is particularly challenging because each member of this genus can possess a varying number of canonical QSS both in its chromosome and/or in its plasmids. What further complicates the analysis is that the genomes of these organisms can contain a large number of LuxR solos, which is usually greater than the canonical systems present in a given strain. An additional complication is that LuxI/LuxR-like proteins display a great variation in their sequences. To address these unknowns, this study was performed with the following objectives: first, to assess the diversity and genetic contexts of *luxI*-like and *luxR*-like genes in *Rhizobium*, focusing on canonical systems to understand their genetic variability and to infer the evolutionary and functional implications of the diversity found; second, to determine whether these genes are encoded on plasmids or chromosomes; third, to examine the extent of coevolution among canonical systems; and fourth, to evaluate the role of horizontal gene transfer in shaping the evolution of these QS systems.

## Methods

### *Rhizobium* genomes and their taxonomic assignment

In December 2023, we downloaded all available complete and closed RefSeq sequences from NCBI classified into the genus *Rhizobium*. We retained 142 assemblies with a level of contamination lower than 5% and a completeness equal to or greater than 95%, and we analyzed these sequences using CheckM (Parks et al., 2015). To check for and correct potential taxonomic discrepancies, we added to our collection the sequences for the 93 *Rhizobium* reference strains deposited in NCBI (Supplementary Material Table S1, available in the online Supplementary Material) and then grouped all strains that shared an average nucleotide identity (ANI) of 95% and a query coverage of at least 70% bidirectionally (Pritchard et al., 2016). With few exceptions, in clusters containing a reference strain, all members were renamed to match the species name of that reference. For clusters with two reference strains, we selected the species name that was described and published first.

Genomes that did not initially group with a cluster were assigned to one of the preexisting clusters if they had an ANI of 95% and at least 70% coverage with at least one member of that particular group. Genomes that clustered with each other with the abovementioned coverage and sequence identity parameters but without the presence of a reference strain were classified as tax_group. Finally, we classified the genomes that did not cluster with any other genome as *Rhizobium* spp.

### Plasmid classification

To classify the plasmids in our collection set, we calculated the ANI of all potential plasmid pairs. Then, we sorted the plasmids in two stages. First, we grouped those plasmids that shared at least 95% nucleotide identity with a sequence coverage of at least 75%. Additionally, we identified plasmids belonging to the *repABC* plasmid family through a BLASTp search, using the RepA (WP_011053501), RepB (WP_004672729.1), and RepC (WP_011053503) proteins encoded on plasmid pRetCFN42d (pSym of *R. etli* CFN42) as queries, with an e-value cutoff of ≤10^−5^ (Ceval et al., 2008). Afterward, with the aim of recognizing symbiotic plasmids, we performed BLASTp searches using NifH (WP_004675840.1) and NodA (WP_004679687.1) of pRetCFN42d as queries, with an e-value cutoff of ≤10^−5^. The results were refined by confirming the presence of the corresponding Pfam-A domains (Fer4_NifH and NodA). Finally, to identify potentially conjugative plasmids, we first identified the Pfam-A domains of each of the proteins encoded by the *trb* and *tra* operons included in the Pfam-A database (Mistry et al., 2021; Supplementary Material Table S2) for the pRetCFN42a plasmid of *Rhizobium etli*, a plasmid that has been experimentally determined to be conjugative (Ding and Hynes, 2009; Tun-Garrido et al., 2003). These domains were subsequently used to scan the proteomes encoded by the plasmids. In addition, the TrbH, TrbJ, and TrbK proteins were searched using BLASTx (e-value ≤10^−5^) because in some cases, the Pfam-A domain was not found or in other cases, there were no available matrices describing the protein, possibly because of its small size. Additionally, if either a *luxI-*like or *luxR-*like gene was located within the genomic neighborhood of the *tra* or *trb* operons, the locus was classified as a potential QS–regulated conjugative system.

### Identification of probable orthologs

The identification of probable LuxI orthologs was conducted in two stages. At the first stage, we performed a BLASTp search (Camacho et al., 2009) using an e-value cutoff of ≤10^−5^. As queries, we employed the protein sequences WP_099058095.1 (*Rhizobium* sp. ACO-34A), WP_074069425.1 (*R. gallicum*, IE4872), WP_003541559.1 (*R. leguminosarum* Vaf-108), and WP_043160651.1 (*Bradyrhizobium* sp. Ai1a-2), share sequence identities between 34.65% and 40%. In the second stage, we refined the set of LuxI-like candidates identified by BLAST in *Rhizobium* species by confirming the presence of the conserved Pfam-A domain PF00765 (Autoind_synth), which is characteristic of LuxI proteins, and the conservation of the domain architecture of genes encoding proteins within their genomic context (GCO). Protein architectures were defined as combinations of one or more nonoverlapping Pfam-A domains that together covered the maximum possible length of a protein (Soto-Avila et al., 2021; Godínez-Pérez et al., 2023). To ensure consistent protein and Pfam annotation, the genome sequences of *Rhizobium* species were reannotated using Prokka v1.14.6 (Seemann, 2014). We then identified protein domains across all the predicted protein-coding genes using the Pfam-A database (Mistry et al., 2021). Domain predictions were performed using HMMER’s hmmscan tool (El-Gebali et al., 2019), with parameters of -E 0.001, -domE 0.1, and -max to ensure full sensitivity and avoid heuristic filters.

Because we were specifically interested in LuxI-like proteins that potentially act in concert with LuxR-like regulators, we examined the GCO surrounding each of the LuxI candidates. For each GCO, we extracted Pfam-A annotations from the 15 neighboring genes to assess the presence of a LuxR-like gene, presenting the following two characteristic Pfam domains found in experimentally characterized LuxR-like proteins: an N-terminal autoinducer-binding domain (PF03472, Autoind_bind) and a C-terminal helix-turn-helix DNA-binding domain (PF00196, GerE) separated by a short linker region. These two domains shape the LuxR-like protein architecture. Finally, to confirm the LuxR-like proteins within these GCOs, we conducted a BLASTp search using LuxR proteins from *Bradyrhizobium* sp. SZCCHNS1054 (WP_316189892.1), *Rhizobium phaseoli* N771 (WP_029531407.1), and *Agrobacterium fabrum* C58 (WP_010974900.1) as queries. Additionally, since orthologs often retain conserved genomic neighborhoods, we clustered LuxI candidates on the basis of GCO similarity to identify shared gene arrangements.

### Grouping genetic contexts

LuxI-like proteins were first identified using BLASTp searches and confirmed by the presence of the corresponding Pfam-A domain. For each *luxI*-like gene, we searched for genes encoding LuxR-like proteins within a genomic window of 15 genes upstream and 15 genes downstream. For loci associated with plasmid conjugative transfer, this window was expanded to accommodate larger, more variable gene neighborhoods.

Based on the Pfam-A domain architectures of all proteins encoded within these genomic contexts, we constructed presence–absence matrices describing the domain composition of each *luxI*-like genetic context. These matrices were subsequently curated manually to resolve alternative protein architectures representing the same contextual protein.

Protein architectures were defined as non-overlapping combinations of domains that maximized coverage of each amino acid sequence. Initially, all domains with an expectation value (E-value) < 0.001 were retained. When predicted domains overlapped, overlaps were resolved by selecting the domain with the lowest E-value that spanned the largest possible fraction of the sequence. The resulting combination was defined as the representative protein architecture for that protein (Soto-Avila et al., 2021; Godínez-Pérez et al., 2023). In cases where contextual orthologs exhibited highly similar domain architectures, BLAST sequence similarity searches were performed to confirm that these architectures corresponded to orthologous proteins with equivalent functions within the context.

With the resulting binary matrix, we constructed a hierarchical similarity dendrogram and calculated distances between the *luxI*-like genetic contexts using the Jaccard index. We performed hierarchical clustering using the UPGMA method with the Scipy package (Virtanen et al., 2020). We converted the resulting structure to Newick format to generate a dendrogram using the ETE3 package (Huerta-Cepas et al., 2016), and we re-rooted the tree using the genetic context of the QS system of *B. diazoefficiens* USDA 110 as an outgroup. We automatically calculated the optimal distance threshold for the dendrogram that maximized the silhouette score (silhouette_score from scikit-learn, https://jmlr.csail.mit.edu/papers/v12/pedregosa11a.html). With this threshold, we generated clusters of genetic contexts using linkage and fcluster (Virtanen et al., 2020). Some of the clusters were curated manually.

### *Rhizobium* species tree

For phylogenetic analysis, we used the complete genome sequence of *Bradyrhizobium diazoefficiens* USDA 110 (GCF_001642675.1) as an outgroup and used the 142 complete *Rhizobium* genomes that we downloaded from NCBI. The pangenome was estimated using Roary v3.13.0 (Page et al., 2015), with a threshold of 80% identity applied for ortholog clustering. The analysis identified 331 core genes, which were used for phylogenetic reconstruction. The multiple alignment was performed with Roary with these core genes, and a maximum likelihood phylogenetic tree was constructed using IQ-TREE v2.0.7 (Minh et al., 2020). The chosen model was TIM2+F + R6 according to the BIC. The robustness of the tree was evaluated using 1,000 ultrafast bootstrap replicates. The resulting tree was visualized via the iTol tool (Letunic and Bork, 2021).

### Phylogenetic reconstruction of LuxI-like and LuxR-like proteins

In this study, we only analyze the sequences of LuxI-like/LuxR-like proteins belonging to canonical QSSs, i.e., those pairs whose genes are located next to each other or separated by only a few genes. We exclude the so-called LuxR solos due to their number, the variety of genetic contexts in which they are found, and the diversity of potential functions they may have, which will be described in a second paper. The selected sequences were aligned with MUSCLE 5 (Edgar, 2004) and trimmed with TrimAI on the basis of default parameters (Capella-Gutiérrez et al., 2009). To construct the tree, we utilized IQ-TREE multicore version 1.6.12 for 64-bit Linux (maximum likelihood). The chosen model was JTT + F + I + G4 according to BIC. To assess branch support, 1,000 ultrafast bootstrap replicates were performed using IQ-TREE v2.0.7 (Nguyen et al., 2015). We selected the AND86768.1 protein for LuxI-like proteins and the AND86767.1 protein for LuxR-like proteins, both from *B. diazoefficiens* USDA 110, as a tree outgroup. The trees, which contained 340 grouped sequences for LuxI and 319 for LuxR, were visualized using the iTol tool (Letunic and Bork, 2021).

### Root-to-tip distance analysis

Root-to-tip distances were computed from the phylogenetic trees of LuxI-like and LuxR-like proteins using the ape package in R (Paradis et al., 2019). To enable comparisons, the trees were constructed using matching taxon identifiers. In cases where a single LuxI-like protein was associated with two LuxR-like proteins or a single LuxR-like protein was associated with two LuxI proteins, each combination was assigned a unique identifier. For each protein family, three separate trees were analyzed: one including only plasmid sequences, another comprising chromosomal sequences and a third containing all sequences. The trees included 107 chromosomal taxa and 216 plasmid taxa. All the trees were rooted on the basis of *B. diazoefficiens* USDA 110 sequences as the outgroup. The distributions of the *root-to-tip distances* were then compared between the chromosomal, plasmid, and overall datasets.

### Analysis of coevolving LuxR–I pairs

To evaluate whether the phylogenies of LuxR-like and LuxI-like proteins are similar, we first computed the normalized Robinson–Foulds (nRF) distance using the R packages ape (Paradis et al., 2019) and phangorn (Schliep, 2011). In the second step, we analyzed the congruence between the two trees and pinpointed putative codiverging pairs. For this, we applied the Procrustean Approach to Cophylogeny (PACo) method (Balbuena et al., 2013). Patristic (cophenetic) distances were obtained with cophenetic (Parad et al., 2004), yielding the matrices D_H (LuxI) and D_P (LuxR) indexed by identical tip sets. Because we named the tree tips with the same names in our analysis, we defined an identity association (A), where each LuxI tip was linked to the LuxR tip of the same name (a binary matrix with values of 1 for matched pairs). We combined D_H, D_P, and A with prepare_paco_data() (paco) and applied Cailliez’s correction via add_pcoord (correction = “cailliez”) to ensure Euclidean embeddability prior to Procrustes superimposition. We then implemented PACo with 1,000 permutations (nperm = 1,000, seed = 42, method = “r2″). Significance was assessed by permutation (p value = proportion of randomized associations with fit ≥ observed); a small p value indicated overall cophylogenetic congruence.

### Link-level residuals and classification

We extracted interaction residuals with residuals_paco (paco_result$proc, type = “interaction”) to quantify local agreement for each LuxI–LuxR pair. Lower residuals denote well-fitting links (consistent with local codivergence), whereas higher residuals indicate incongruence (e.g., host switching, paralogy/recombination, rate heterogeneity, or labeling/mapping errors). For interpretation, we used data-driven quantile bands from our dataset: Low < Q1 (∼0.38); Medium = Q1–Q3 (∼0.38–1.14), reflecting the moderate mismatches expected from ordinary differences in topology/branch length, noise, or limited resolution; and High ≥ p90 (∼1.35). Extreme outliers were defined as those with a residual greater than Q3 + 3 × IQR (∼3.42). The results of this analysis were plotted using the R package ggplot and in a mirror tree (tanglegram) visualized with iTOL (Minh et al., 2020).

## Results

### *Rhizobium* taxonomy

The initial species tree was established via the taxonomic assignments provided by the authors who submitted the sequences to NCBI. The resulting phylogeny revealed multiple inconsistencies, with species dispersed across clades incongruent with their presumed taxonomy. In addition, we often observed that within a clade, different species were tightly grouped together. To correct this problem, we decided to rename the species as described in the Methods section. The tree we constructed using these new taxonomic assignments shows consistent clades. The tree encompasses 35 *Rhizobium* species, 23 *Rhizobium* spp.*,* and four genome clusters without a reference strain, which we named tax_groups A, B, C, and D. We also identified four genome clusters that met our criteria for sequence identity and coverage, containing at least two NCBI reference strains. For the strains grouped with *R. gallicum* and *R. mongolense,* we opted for the species name *R. gallicum,* considering that it was described 1 year earlier (Amarger et al., 1997; van Berkum et al., 1998).

For the strains that shared the same clade with *R. favelukesii* and *R. tibeticum,* we chose *R. tibeticum* as the species name for this group because it was described several years earlier (Torres et al., 2016; Hou et al., 2009). In the third case, strains in the same cluster as *R. acaciae* and *R. johnstonii* were renamed as belonging to *R. johnstonii* (Hsouna et al., 2023; Young et al., 2023). Finally, one strain clade contained three NCBI reference strains: *R. leguminosarum, R. beringeri*, and *R. indigoferae.* However, Menéndez and coworkers reclassified *R. indigoferae* into *R. leguminosarum,* and for this reason, we used this taxonomic assignment (Menéndez et al., 2024). We kept the name *R. beringeri,* considering that the complete genomes of this species form a separate and consistent clade, as observed by Young and coworkers (Wei et al., 2002; Young et al., 2023). If a strain had a previous species name but did not group with any NCBI reference strain or with any other genome, it was renamed *Rhizobium* sp. In the phylogenetic tree, we marked those strains whose species name within *Rhizobium* was modified with a single asterisk (*).

**FIGURE 1:**
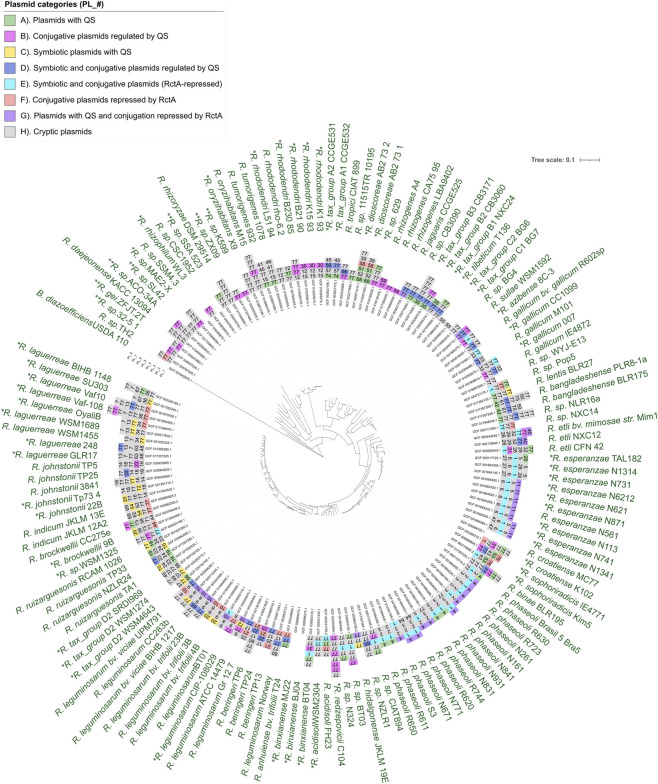
*Rhizobium* species maximum likelihood phylogenetic tree. The tree was inferred from 142 strains using *Bradyrhizobium diazefficiens* USDA 110 as an outgroup, based in the concatenated alignment of 331 core genes. The external circle shows the species and strain names used in the tree. Strains marked with an asterisk (*) were renamed in this analysis, and those marked with a double asterisk (**) are now members of other genera close to *Rhizobium*. The inner circles indicate the number of plasmids of each strain; one square, one plasmid. The number within each square shows the plasmid group and the color, its category. The most internal circle shows the assembly accession numbers.

We must note that nine species classified as *Rhizobium* when we downloaded the genomes are now members of other genera closely related to *Rhizobium*, such as *Pararhizobium* (Mousavi et al., 2015), *Peteryoungia* (Rahi et al., 2021), and *Neorhizobium* (Mousavi et al., 2014). Although we retained their previous names, we marked these strains with a double asterisk (**) in the phylogenetic tree to indicate their updated genus-level classification*.* The only purpose of all these changes was to simplify our analysis, and the complete list of modifications is provided in Supplementary Material Table S3. To reveal the diversity of the genus and relationships among the *Rhizobium* species, we constructed a maximum likelihood phylogenetic tree based on the core genome genes (Figure 1).

### Canonical QSSs and their genetic context on chromosomes

In general, the genomes of members of the *Rhizobium* genus contain only one chromosome, but a few also contain a secondary chromosome or a chromid. Among our collection of 142 *Rhizobium* members, 12 exhibited this phenomenon.

**FIGURE 2:**
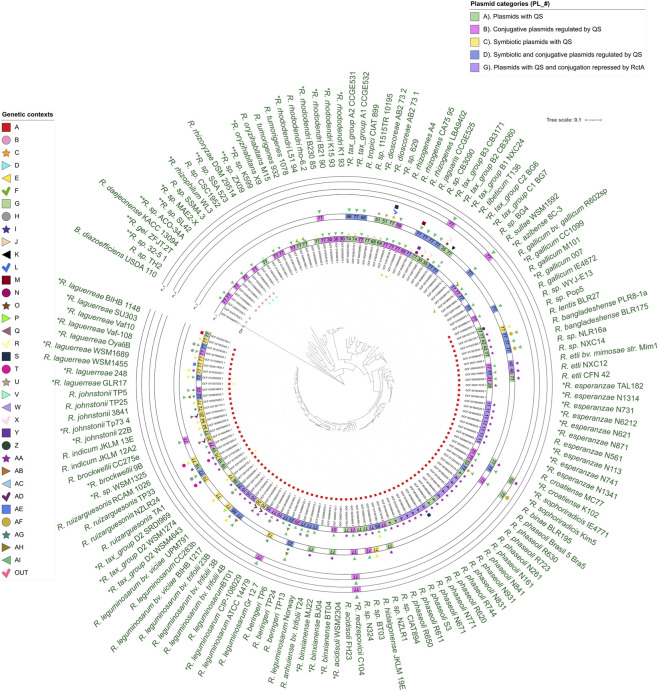
Locations of the QSS and their genomic contexts are mapped in the phylogenetic tree of Rhizobium species. The figures and colors in most internal circles (Chr) indicate which chromosome encodes a QSS and the genomic context in which it occurs. Squares in the external circles (PL_1 to PL_4) show the plasmids encoding QSS, their plasmid group (number inside the square), and their category (color). The figure above each plasmid square shows the QSS genomic context for that plasmid. The assembly accession numbers are also shown.

Canonical quorum-sensing systems (QSSs) were identified on 102 of the 154 *Rhizobium* chromosomes analyzed, with five genomes harboring two chromosomal systems. Thirty-one strains lacked chromosomal QSSs but carried plasmid-encoded systems, and these strains clustered together in the species phylogeny. Nine strains lacked any canonical QSSs: *R*. sp. BG4, *R. tax_group C1 BG7, *R. tax_group C2 BG6, *R*. *rhizoryzae* DSM29514, *R. *oryzihabitans* X9, *R. rhododendri L51 94, *R. mongolense CC1099, *R*. sp. CSC1952 and *R. gallicum 007. These strains were distributed across different clusters in the species’ phylogenetic tree (Figure 2), indicating independent losses.

To explain the observed distribution, two scenarios may be invoked: either the ancestral *Rhizobium* chromosome encoded a QSS that was subsequently lost in multiple lineages, or canonical systems were acquired independently on several occasions. Genomic context (GCO) analysis supports the latter, as chromosomal QSSs are embedded in seven distinct contexts (A–G), rather than a single conserved configuration (Supplementary Material Figure S1; Supplementary Material Table S4). Because many genes surrounding *luxI*- and *luxR*-like loci encode proteins of unknown function, we used Pfam-based protein domain architectures (López-Guerrero et al., 2012) to identify these contextual genes and confirm the identity of *luxI/luxR* homologs. This approach enabled a robust classification of chromosomal contexts.

Context A was the most prevalent, encompassing 91 strains and including seven conserved flanking genes. Two with conserved unknown functions (DUF4174 or DUF2934 domains) and others encoding defined functions such as SBP_bac_8, a GntR-family transcriptional regulator, and a response regulator. LuxI- and LuxR-like proteins in this context exhibited >95% sequence identity, and strains carrying context A clustered together in the species tree, except for *R. laguerreae* BIHB1148, which lacks a chromosomal QS system.

Contexts B–G (data shown in Supplementary Material Figure S1), were less common and more phylogenetically restricted. Context B appeared in several *Rhizobium* species and displayed similarity to the *ngrI/ngrR* system of *Sinorhizobium fredii* NGR234. Context C was associated with chromids. In the case of context E, formed part of a symbiotic island containing nodulation and nitrogen-fixation genes like *nodO* and *nifHDK*. Contexts F and G were unique to *R. azibense* 8C-3 and *Rhizobium* sp. TH2, respectively; context F also carried T4SS-related genes consistent with remnants of an integrative conjugative element (ICE) (Rajnovic et al., 2022; Cordeiro et al., 2017).

Strikingly, strains containing chromosomal QSSs outside of context A grouped phylogenetically with strains lacking chromosomal QS, and in the few genomes with two QSSs, one system consistently belonged to context A while the second resided in a distinct context. These patterns collectively support multiple, independent acquisitions of QSSs across *Rhizobium* evolution.

### Plasmid classification

With few exceptions, the *Rhizobium* genomes contained a wide range of very large plasmids, with up to eight in select strains. The collection contained 574 plasmids, most of which included at least one *repABC* operon necessary for plasmid replication and maintenance (Ceval et al., 2008). We classified them according to their ANI into 76 groups. The largest, named Group 1, contains 34 members; Group 2 and Group 4, each have 15 members. In contrast, 39 groups consist of only two members, and 256 plasmids were orphans (singletons); all of which were grouped together in a heterogeneous set referred to as Group 77.

Mapping these groups onto the species phylogeny revealed that Group 1 is widely dispersed across species (i,e., *R. etli, R. phaseoli, R. croatiense, R. esperanzae, R. azibense,* and *R. leguminosarum* bv*. phaseoli*), especially among strains isolated from nodules of bean plants (*Phaseolus* spp.) (López-Guerrero et al., 2012; Rajnovic et al., 2022; Cordeiro et al., 2017; Kaur et al., 2023; Shamseldin et al., 2022; Mora et al., 2014; Martinez-Romero et al., 2024). Some plasmids groups (i.,e., Group 1, 15, 17, 20, 21, 22, 25, 31, 60, 65, 66 and 72) are found in multiple *Rhizobium* species, while others are confined to a single *Rhizobium* species (for instance, Groups 2–4 restricted to *R. phaseoli,* Groups 8–10 in *R. leguminosarum,* Groups 5 and 11 in *R. esperanzae*). As expected, the orphan plasmids are widely distributed in the phylogenetic tree. Some *Rhizobium* strains possess only orphan plasmids (Figure 1).

**FIGURE 3:**
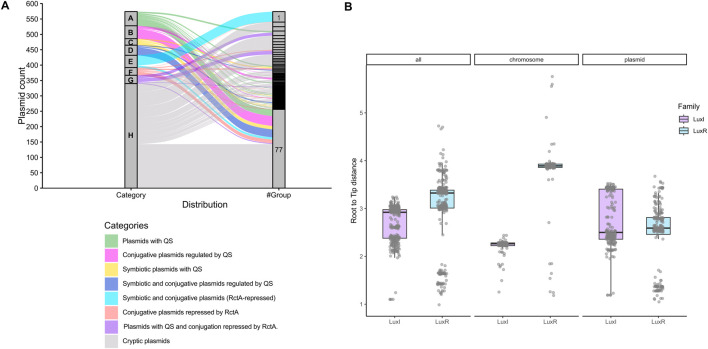
**(A)** Plasmid groups and their categories. This graphic links the plasmid groups 1 to 77 (column on the right) with the plasmid categories (A–H) they contain (column on the left). **(B)** Root-to-tip phylogenetic distances. Boxplots of the root-to-tip phylogenetic distances of LuxI-like and LuxR-like proteins encoded in chromosomes and plasmids (combined and separated).

We also classified the *Rhizobium* plasmids into eight categories (A-H) based on traits such as the presence of symbiosis-related genes, QSS, and conjugative transfer regulated by QS or repressed by RctA. Categories A-D and G include plasmids carrying QS systems, with varying combinations of conjugative and/or symbiotic traits. Categories E and F consist of symbiotic or conjugative plasmids that lack QS. Category H contains 340 plasmids that do not fit into any of the categories defined above and which we call cryptic plasmids. Orphan plasmids (Group 77) are represented in any of these categories. In Figure 3A, we show the relationships between plasmid groups and plasmid categories.

**FIGURE 4:**
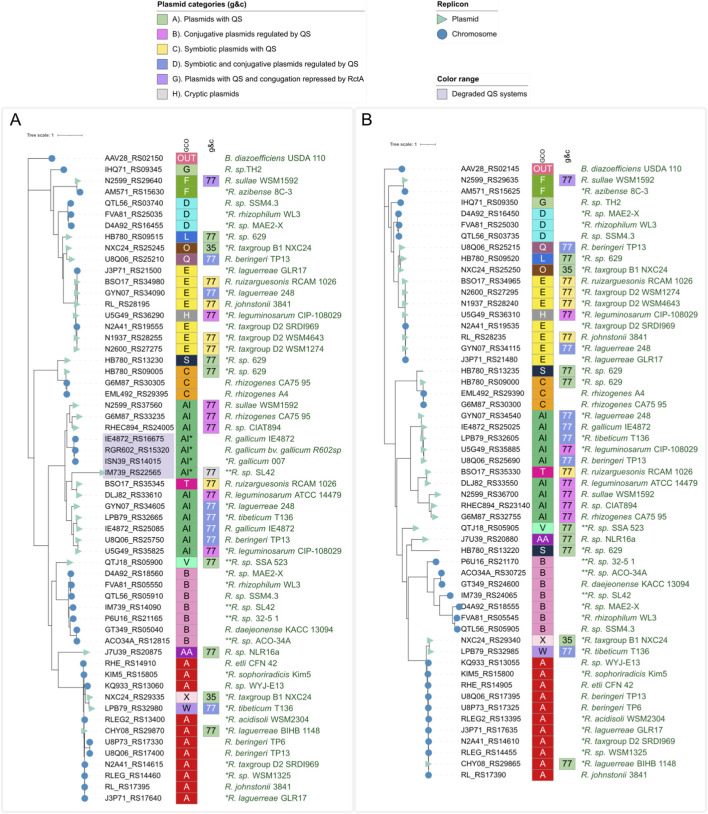
**(A)** LuxI-like Maximum likelihood phylogenetic tree of chromosomal and plasmid LuxI-like protein. Labels on the tree indicate if the proteins are encoded in a plasmid or a chromosome. The most external boxes circle indicates the plasmid group, and the following boxes circle show the plasmid category. **(B)** LuxR-like Maximum likelihood phylogenetic tree of chromosomal and plasmid LuxR-like protein. Labels on the tree indicate if the proteins are encoded in a plasmid or a chromosome. Labels on the tree indicate if the proteins are encoded in a plasmid or a chromosome. The outer boxes show plasmid information, where numbers indicate the plasmid group and colors indicate the plasmid category (G&C). Inner boxes indicate the genomic context (GCO).

### QSSs located on plasmids

In *Rhizobium*, plasmids carrying QSSs are highly diverse in terms of both: how many QSS-bearing plasmids appear per strain or per plasmid and the types of plasmids involved. In our search, we found 207 QSSs distributed across 169 plasmids belonging to 119 strains. In 59 of them, we found that only one of their plasmids contains QSSs. Most of these plasmids had only one QSS; however, we found 15 plasmids with two QSSs and three strains with a plasmid with three QSSs. In contrast, we also found strains with more than one plasmid containing one or more QSSs. The record is held by the strain *R. redzepovicii C104, which contains 4 plasmids encoding QSSs (Supplementary Material Table S5). Nonetheless, we found 18 strains without QSSs encoded in plasmids. In our analysis, we found four QSSs with *luxI*-like duplications and five with *luxR*-like duplications.

### QSSs located on plasmids and their genomic contexts

The 207 QSSs found on *Rhizobium* plasmids are embedded in 32 different genomic contexts, which we designated as follows: A, C, E, F, H-Z, and AA to AI (Figure 2). We were surprised to find that the chromosomal contexts A, C, E, and F, described above, were also present on plasmids. For example, Context E, containing two chromosomal QSSs, was also found on 14 plasmids. Context F was found on the chromosome of strain *R. azibense 8C-3 and on three plasmids. These observations reinforce the hypothesis that information is exchanged between plasmids and chromosomes (All information on the chromosomal and plasmid GCO, as well as their plasmid classification by group and category, is shown in Supplementary Material Table S6, and the hierarchical similarity dendrogram of the genetic contexts is shown in Supplementary Material Figure S2).

The most prevalent plasmid context, designated AI, is found on 75 plasmids, all of which are conjugative or potentially conjugative and contain the *traI* (*luxI*-like)/*traR* (*luxR*-like) genes and the *tra* and *trb* operons. 63 strains possess at least one conjugative plasmid regulated by QS. Of the 75 potentially conjugative plasmids regulated by QS (category B), 33 are also symbiotic (category D). Most of these plasmids belong to Group 77. In the operons involved in conjugation, we observed six distinct gene-organization types (Supplementary Material Figure S3).

The second-largest context, designated AA, is found on 51 different plasmids, each from a different strain. This context is characterized by the presence of the *raiI*/*raiR* genes, along with *dadR,* an LRP-family regulator controlling adjacent alanine-metabolism genes (Wisniewski-Dyé et al., 2002).

The AA context is found on one plasmid in all strains of *R. phaseoli, R. etli, R*. *ruizarguesonis, R. bangladeshense, R. sophoriradicis*, and sporadically on plasmids of other species (Supplementary Material Figure S4). Among the 51 plasmids containing this context, 25 belong to category G (plasmids with QS and conjugation repressed by RctA), 25 to category A (plasmids with QS), and only 1 to category C (symbiotic with QS).

Context E is widely distributed within 14 symbiotic plasmids: 11 in category C and 3 in category D. Notably, this context is also found on two chromosomes. This context is characterized by a *rhiABCD* operon flanked by *rhiI* (a luxI-like gene) at one end and *rhiR* (a luxIR-like gene) at the other. The RhiB protein contains a Pfam-A domain, classified as a phage tail collar domain, and may be involved in plant–bacteria interactions (Cubo et al., 1992). However, no Pfam-A domain was found in the RhiA and RhiC proteins, but a BLASTx search was performed using the equivalent sequences from plasmid pRle248a as a query, and they were found to be conserved across all members of this context.

The rhiI–rhiABCD–rhiR cluster, typically flanked by *nod* and *nif* genes, was detected in all strains of *R.* tax_group D2 and *R. indicum*, in five of 9 *R. laguerreae* strains, and in a single representative of *R. leguminosarum*, *R. johnstonii*, *R. ruizarguesonis*, *R. hidalgonense*, and *R. lentis* (Supplementary Material Figure S5).

Context I is present in two plasmids of the four strains of *R. ruizarguesonis*. Context H is particularly interesting because we identified this context not only on symbiotic plasmids but also on plasmids with other functions. This context is present on three plasmids of the 11 strains of *R. leguminosarum*, on two of the nine strains of *R. laguerreae*, and on only one plasmid of the 4 *R. ruizarguesonis* strains. Finally, the M context is found only on the pRCCGE525b plasmid of *R. jaguaris* CCGE525, which also shares genes with the AA context (Supplementary Material Figures S4,S5).

Contexts A, AB, AC, AE, C, K, L, M, N, P, R, V, W, Y, and Z were each detected on a single plasmid, all belonging to Group 77. With the exception of the highly prevalent contexts AI, AA, and E, all remaining contexts were restricted to between two and six plasmids.

### QSSs and transposition

Transposition is a common way of transferring DNA from one site in the genome to another, or with the help of conjugative plasmids, from one strain to another. To assess the potential role of transposition in the evolutionary dynamics of QSSs, we searched for the presence of genes encoding transposases in the genomic contexts of QSSs, especially those bordering *luxI*-like genes, *luxR*-like genes, or both.

In contexts E, H, and AF, we identified one plasmid per context carrying a *luxI/luxR*-like gene pair flanked by genes encoding proteins with the HTH_Tnp_1 transposase domain (PF01527). Additionally, two plasmids belonging to context H were found in which the *luxI*-like gene was bordered by genes encoding a transposase with the same domain. However, members of context H lacking associated transposase genes were also observed. In all plasmids assigned to context I, the *luxI*-like gene was consistently flanked by a gene encoding an HTH_Tnp_1 transposase. Beyond these cases, we identified 29 additional plasmids containing a single gene encoding the same transposase type within their QSS contexts, as well as two chromosomal QSS contexts displaying similar features.

In contexts P and W, *luxI/luxR*-like genes were flanked by genes encoding a DDE_Tnp_IS240 transposase (PF13610). A *luxI*-like gene bordered by this transposase was also detected in a plasmid belonging to context AI. Furthermore, three additional members of context AI and one member of context AB each contained a gene encoding this transposase. Genes encoding proteins containing the DEDD_Tnp_IS110|Transposase_20 domains (PF01548|PF02371) were identified on the chromosome of *R. esperanzae* TAL182, flanking its QSS (context A). A single transposase gene was found adjacent to QSS genes in another chromosomal context A and in six plasmids representing diverse QSS contexts.

Taken together, these observations strongly suggest that transposition may play a significant role in shaping the genomic distribution and evolutionary dynamics of QSS genes.

### Degraded QS systems

To identify the QSSs whose LuxI/LuxR-like genes are suspected of no longer being functional and that could be in the process of disappearing, we reviewed both the annotations reported in NCBI and those that we obtained, searching for pseudogenes, truncated genes or the complete loss of either *luxI*-like or *luxR*-like genes in different genomic contexts.

We identified *luxI*- or *luxR* pseudogenes in both chromosomal and plasmid contexts. Regarding the chromosomes, we found *luxI*-like pseudogenes in the strains *R. beringeri* TP13, *R. beringeri* TP6, and *R. johnstonii* Tp73. We also detected *luxI*-like pseudogenes in plasmids of the AI context: unnamed2 of **R. sp. 32–5 1 and pRspBT03a of *R. sp*. BT03.

Our analyses also revealed seven genes encoding extremely small LuxI-type proteins, ranging in size from 42 to 162 aa. This size suggests that these proteins are no longer functional; however, they retain the Pfam-A domain of Autoind_synth. These genes appear in incomplete or orphan genomic contexts.

We found a unique case in the pRlX5 plasmid of *R. beringeri* TP6 that, on the one hand, lacks a gene encoding a LuxR-like protein but, on the other hand, encodes a translational fusion of a LuxI-like protein with a peptide containing a Pfam-A WGR domain. This fusion is located adjacent to a *repABC* operon.

We identified AI-genetic contexts that are almost complete but lack a *luxI*-like gene in plasmids pRL8, unnamed4, unnamed3, and pCC283b_5 from *R. johnstonii* 3,841, *R. laguerreae* Vaf10, *R. laguerreae* Vaf-108, and *R. leguminosarum* CC283b, respectively. In some cases, the Mpf genes are distant or absent, and the *luxI*-like gene is missing, yet the Dtr region remains close to a *luxR*-like gene. On the other hand, we observed plasmids that retain a *luxI*-like gene but exhibit degenerated conjugation modules and lack a *luxR*-like gene. All data regarding the degraded QS systems section are included in Supplementary Material Table S7.

### Phylogenies of LuxI-like and LuxR-like proteins

To evaluate the diversity of LuxI-like and LuxR-like protein sequences in relation to the genetic compartment in which the genes encoding them are located, we first estimated maximum likelihood phylogenies of all LuxI-like proteins encoded on plasmids and phylogenies of those whose genes are located on chromosomes. We constructed an integrative tree of all these proteins. We followed the same strategy for LuxR-like proteins. To determine whether LuxI-like or LuxR-like proteins are more diverse, we evaluated the root-to-tip distance of each of these trees and observed the following trends: the LuxI-like phylogeny trees have shorter distances than their LuxR-like counterparts. We also observed that the root-to-tip distances for the phylogenies of LuxI-like and LuxR-like proteins encoded on plasmids are greater than those of their chromosomal counterparts (Figure 3B). Notably, the root-to-tip distances of the LuxI-like and LuxR-like trees are much shorter than those of the trees for other chromosomal genomic contexts. These observations indicate that QSSs encoded on plasmids are much more diverse than chromosomal QSSs and that LuxR-like proteins are more diverse than LuxI-like proteins are.

### Coevolving LuxI and LuxR pairs

To evaluate potential coevolution between LuxI-like synthases and LuxR-like receptors, we compared their phylogenies (Supplementary Material Figure S6) by correlating cophenetic distance matrices (Parad et al., 2004). The high correlation (r = 0.85) indicated broadly parallel evolutionary histories, although approximately one-quarter of the pairs deviated from the main trend. PACo analysis (Balbuena et al., 2013) confirmed a significant global fit (permutation p < 0.001), consistent with widespread but not universal codivergence.

PACo residual classes showed a clear association with genomic location and context. Most chromosomal LuxI–LuxR pairs fell within the low-residual class and clustered in a single clade shared by both trees, corresponding to conserved genomic context A and resembling canonical CinI/CinR systems (pairs listed in Supplementary Material Table S8; see residual distribution in Supplementary Material Figure S7). In contrast, high and extreme residuals were predominantly associated with plasmid-encoded LuxR-I systems. All four extreme outliers were plasmid-borne, including *R. rhododendri* B230 85, *R. gei* ZFJT.2T, *R. oryzihabitans* M15, and *R*. sp. 629, frequently found on group-77 conjugative plasmids, indicating non-codivergent histories.

Intermediate residuals were common among plasmidic pairs distributed across multiple genomic contexts (AA, AI, X, Y, W). For example, context-AA plasmids from *R. phaseoli* and *R. esperanzae* formed a cohesive clade despite moderate residuals, whereas context-AI plasmids showed heterogeneous residual values and greater topological discordance, as illustrated by *R. gei* ZFJT.2T and *R. oryzihabitans* M15, presenting extreme residual values. Detailed strain-level assignments for all residual classes are provided in Supplementary Material Table S8.

Overall, plasmid-encoded systems exhibited extensive context mixing and paralogy, whereas chromosomal systems remained largely context-stable. In total, 28 of 33 high- and extreme-residual pairs were plasmid encoded, underscoring the greater evolutionary instability of plasmid-borne quorum-sensing systems relative to their chromosomal counterparts.

### Plasmids and Rhizobium chromosomes can exchange QSS genes

As we described above, genomic contexts A, C, E, and F are present not only on *Rhizobium* chromosomes but also on some plasmids, suggesting that QSS genes are exchanged between plasmids and chromosomes in *Rhizobium*. To assess the potential role of *Rhizobium* plasmids as donors of QSS genes to the chromosomes of this genus, we constructed phylogenetic trees using LuxR-like and LuxI-like sequences encoded either on chromosomes or on plasmids, with the WAG + F + I + G4 model selected according to BIC. In these trees, we observed that some LuxI-like/LuxR-like sequences encoded on plasmids were closely related to proteins that are encoded on chromosomes. To illustrate this finding, we show representative trees in Figures 4A, B. For example, LuxI/LuxR-like products of the symbiotic plasmids of the *R. johnstonii* 3,841 and *R.laguerreae 248 strains are grouped with the equivalent products of the chromosome of strain *R.laguerreae GLR17. Similarly, the LuxI/LuxR-like proteins encoded on the symbiotic plasmids of strains *R. *leguminosarum* CIP-108029, *R. tax_group D2 WSM1274, and *R. tax_group D2 WSM4643 are grouped with the luxI/luxR-like products of the *R tax_group D2 SRDI969 chromosome. Additionally, the LuxI/LuxR-like products of the conjugative plasmid of *the R. sullae* WSM1592 strain share a clade with the LuxI/LuxR-like proteins encoded on the *R. *azibense* 8C-3 chromosome. In the same way, the two LuxI/LuxR–like products of the orphan plasmid p_1 are located near each other and near the equivalent products of the chromosomes of the strains *R. rhizogenes* CA75 95 and *R. rhizogenes* A4. Finally, the LuxI/LuxR-like proteins encoded on the conjugative plasmids pRheCIAT894b of strain *Rhizobium* sp. CIAT894, pTiCA74_95 of *R. rhizogenes* CA75 95, and pWSM1592_3 of *R. sullae* WSM1592 are grouped with the chromosomal LuxI-like proteins of *R. gallicum* strains 007, IE4872, and R602sp, in which only the *luxI* gene is present, reflecting degraded QS systems in the AI context or remnants of ICE. Interestingly, the *luxI*-like/*luxR*-like genes present on plasmid pSK03 of the *R.laguerreae BIHB 1148 strain are embedded in context A, which is present on many *Rhizobium* chromosomes. These observations indicate that in *Rhizobium,* the QSS genes can be transferred from plasmids to chromosomes and *vice versa*.

## Discussion

In this study, we demonstrated that the distribution of genes encoding canonical QSSs in *Rhizobium* is extremely diverse within and across species. Some strains completely lack these systems, whereas others contain a QSS on the primary chromosome, on a secondary chromosome or on a chromid. Still others encode a QSS on one or more of their plasmids. Finally, there are strains that encode QSSs both on their chromosome and on one or more of their plasmids. This distribution does not appear to have a strong phylogenetic bias, as there are examples of each of these architectures throughout the species tree shown in Figures 1, 2. When we analyzed the chromosomal QSSs, we determined that they are embedded in seven different genomic contexts. Strains that share the genomic context for their QSS are always phylogenetically related. These observations suggest that QSSs located in different genomic contexts were acquired during independent events.

The number of strains sharing a given genomic context varies widely; for example, context A is shared by 91 strains, whereas the other contexts are a characteristic of only one strain. Genomic contexts shared by two or more strains are characterized by having several genes in common; however, some of these contexts also contain genes that are present in some strains but not in others. Differences in the evolutionary paths followed by each genome can be explained by recurrent gain or gene loss. However, we suggest that the genes conserved within a given genomic context could be regulated by the QSS with which they are associated. We suggest that it is precisely these genes that initially gave a selective advantage to the strains that acquired them. Analysis of the transcriptomes of wild-type strains and their mutant derivatives lacking their chromosomal LuxI-like genes under specific conditions should shed light on the contextual genes affected by the mutation, as well as on other important functions such as biofilm formation, nodulation and motility. Additionally, QSSs can be negatively regulated at different levels; some bacteria encode molecules that modulate transcription either through specific regulatory proteins, i.e., RsaL (*Pseudomonas aeruginosa*) (He et al., 2024) or small antisense RNAs (Thomason et al., 2019; Hirakawa et al., 2012), Other organisms modulate the QS response through protein-protein interactions, as is the case with *A. fabrum* (*A. tumefaciens*), in which TraM interacts with TraR to regulate its action (Wetzel et al., 2019). There are also microorganisms that regulate QS responses through Quorum quenching mechanisms (Sikdar and Elias, 2020). These systems are highly varied and can even vary within the same genus. This suggests that these regulatory systems are a secondary adaptation. This is surely an essential element that must participate in the evolutionary dynamics of QSSs in the *Rhizobium* genus.

Plasmids in *Rhizobium* are extraordinarily diverse in terms of sequence, organization, and distribution across the species that make up the genus. The plasmids that encode QSSs belong to many groups and categories, but most QSS genes are located on orphan plasmids, which we grouped here in the miscellaneous group 77. The contexts in which QSSs are found are much more diverse than those of their chromosomal counterparts. Notably, some QSSs are encoded on plasmids classified in different groups and categories, indicating that plasmids can recombine with each other. An example of this phenomenon is plasmids with a *rhiABCD* operon, which is relevant in *Rhizobium*–legume interactions. This operon can be found in different genomic contexts, occurring in 24 plasmids, 18 of which are in group 77, and on two chromosomes, those of *R. laguerreae GLR17 and *R. tax_group D2 SRDI969.

Many plasmids encoding a QSS harbor QS-regulated conjugation genes, while others may also be symbiotic plasmids, suggesting their possible function. However, there are many other plasmids encoding QSSs whose genes provide no insight as to the function of these systems.

The phylogenetic trees of the LuxI-like and LuxR-like proteins reveal some notable properties of the QSSs in *Rhizobium* (Supplementary Material Figure S6). First, the root-to-tip distances of the phylogenetic trees of LuxI-like proteins are smaller, on average, than the root-to-tip distances of the LuxR-like trees, indicating that LuxR-like proteins evolved more rapidly. Second, the root-to-tip distances of the phylogenetic trees of chromosomal LuxI-like and LuxR-like proteins are generally smaller than those of their plasmid counterparts, proving that plasmid-encoded QSSs are more diverse than those encoded on chromosomes. Furthermore, the results of our coevolutionary analysis of LuxI and LuxR-like pairs clearly reveal that chromosomal systems coevolved, in contrast to some plasmid LuxI and LuxR-like pairs, which showed little or no evidence of codivergence.

In this sense, the high degree of coevolution observed between chromosomal LuxI- and LuxR-like pairs points to a stable, long-term evolutionary relationship. These systems are likely preserved through vertical inheritance, evolving in step with the host genome rather than being frequently shuffled by horizontal transfer. This codivergence reflects tight functional coupling, namely, the AHL signals produced by LuxI remain specifically recognized by their cognate LuxR receptors, ensuring compatibility and maintaining the fidelity of quorum-sensing regulation.

In contrast, the low or absent codivergence observed for some plasmid-encoded LuxI- and LuxR-like pairs suggests a far more dynamic and flexible evolutionary path. Plasmids are prone to horizontal transfer across hosts (Redondo-Salvo et al., 2020) a process that disrupts the coadaptation of LuxI and LuxR partners. Consequently, LuxR-like receptors can be recruited in a modular fashion, sometimes pairing with noncognate LuxI-like synthases or even responding to unrelated signals. This reduced coevolution allows plasmid-borne QSSs to exhibit a higher degree of adaptability than chromosome-encoded systems, enabling them to adapt more easily to new genomic contexts and environmental conditions.

QSSs can be lost when they no longer provide a selective advantage to the population. In this study, we identified QSS in which the *luxI-*like or *luxR-*like gene has already disappeared or in which the encoded products are so small that they are likely no longer functional, probably because of the acquisition of frameshift mutations.

In this work, we found clear evidence indicating exchanges of QSS genes between plasmids and chromosomes. First, chromosomal contexts A, C, E, and F are also found in plasmids. Chromosomal context A was also detected on the plasmid pSK03 of *R. laguerreae BIHB 1148. In the phylogenetic trees of representative chromosome-encoded and plasmid-encoded LuxI-like and LuxR-like proteins shown in Figures 4A, B, several LuxI-like or LuxR-like plasmid-encoded proteins are grouped with representative chromosomal LuxI-like or LuxR-like proteins in the same clade. The last evidence of gene exchange is described above: some of the QSSs encoded on plasmids exhibited weak codivergence. This observation could be a consequence of the exchange of luxI- or luxR-like pairs with other plasmids or even with chromosomes.

To explain these QSS distribution patterns, we must assume that we cannot determine the events that occurred before the diversification of the *Rhizobium* genus with respect to QSSs given that, as we have already described, QSSs can be both gained and lost. A brief analysis of the presence of QSSs in other members of the Rhizobiaceae family revealed that they possess these systems, but the systems are embedded in genetic contexts different from those found within *Rhizobium*. One possible explanation for the presence of seven different contexts for QSSs on *Rhizobium* chromosomes is that duplication events of the QSS in one or more Rhizobium ancestors generated paralogs at different positions. In a second event, the ancestral gene was lost, and the different paralogs were preserved in different species of the genus. The other, simpler explanation is that the different QSS systems were acquired and continue to be acquired through various horizontal gene transfer events and that plasmids are the most likely donors.

To assess the role of plasmids in shaping the evolution of QSSs in *Rhizobium*, we must first consider that members of this genus usually contain between one and eight very stable, large plasmids. Second, the universe of plasmids that *Rhizobium* can possess is very large, as evidenced by the fact that 44.5% of *Rhizobium* plasmids are orphan plasmids. This finding also suggests that throughout evolution, members of this genus frequently acquire and lose plasmids. A common observation is that members of a given *Rhizobium* species do not necessarily possess the same plasmid pattern. Third, a significant percentage of these plasmids possess a QSS, a property sometimes linked to symbiotic plasmids, other times to conjugative plasmids, and also to plasmids that cannot be determined to participate in a specific function because the genes they carry are annotated as hypothetical.

These QSSs will remain in the population either because they are essential for the biological functions of the plasmids that carry them (e.g., conjugation or copy number regulation) or because they are important for the bacteria that possess them under certain environmental circumstances (e.g., symbiosis or pathogenesis) (Barton et al., 2020; Pappas, 2008; Li and Farrand, 2000; Miao et al., 2018; Tang et al., 2020; Cervantes et al., 2019). If some of these systems are especially important for a population, individuals will be selected to ensure that these genes are transferred to more stable sites, such as chromosomes. If environmental circumstances change and the acquired QSS no longer confers selective advantages, the system is degraded and eventually lost. The evolution of QSSs in *Rhizobium* is highly dynamic, and plasmid-mediated horizontal transfer plays a crucial role in driving this process.

In this study, we also identified QSSs in which the *luxI-like* or *luxR-like* genes have been lost or whose encoded products are so small that they are likely no longer functional, probably because of the acquisition of a frameshift mutation. Similar patterns of QS system degradation have been reported for the integrative and conjugative elements (ICEs) and integrative mobilizable elements (IMEs) of *Mesorhizobium* (Colombi et al., 2021).

The loss of a *luxI-*like gene or its cognate *luxR-*like partner may provide an opportunity for the recruitment of a new luxI-like/luxR-like member to compensate for the missing counterpart. We suggest that such events could occur as follows: if the incorporated element is a LuxR-like protein, it must satisfy two requirements. First, it should be capable of binding to a broad range of AHLs, as exemplified by TraR (LuxR-like) from the pTiC58 plasmid, which functions as a biosensor for these molecules (Farrand et al., 2002). Second, it must be able to recognize *lux* boxes to act as a transcriptional activator. This binding may initially be weak, but it must then be refined through selection for variants that are more effective in this recognition. In the case of a *luxI-like* element, the AHL synthase must produce AHLs that are recognizable by the corresponding LuxR-like partners.

Finally, we examine how several QS systems can coexist in *Rhizobium*. The simplest answer is that the LuxI-like synthases of a given strain produce autoinducers recognized only by their cognate LuxR-like receptors. However, since it has been shown that *Rhizobium* LuxI-like synthases can synthesize several autoinducers and that LuxR proteins can often respond to many of these molecules, crosstalk can occur. In *Rhizobium*, this issue has been resolved by establishing a hierarchy: one of the systems, usually the one encoded on the chromosome, regulates the expression of the other systems (Zheng et al., 2015; Wilkinson et al., 2002; Danino et al., 2003; Lithgow et al., 2000). This hierarchy is essential for several QSSs to coexist within the same cell without deleterious effects.

## Data Availability

The original contributions presented in the study are included in the article/Supplementary Material, further inquiries can be directed to the corresponding authors.

## Supplementary material

SUPPLEMENTARY FIGURE S1 Gene content of the QSSs genomic contexts encoded in the Rhizobium chromosomes **(A–G)**. Panels show the genes shared in each genomic context (black squares). Blue squares represent LuxR-like genes and the pink ones, the LuxI-like genes. Squares in colors represent genes that are present in more than one genetic context. Names at the right side of each panel list the strains possessing that specific genomic contexts. The numbers above the squares show the index of the genes listed in Supplementary Material Table S4. In context A and B, we included only a few examples of the strain containing in the context. Within the strains of context B, we included the QS of *Sinorhizobium fredii* NGR234 to point out the similarities with this strain and those *Rhizobium* containing QS with context B.

SUPPLEMENTARY FIGURE S2 Hierarchical similarity dendrogram of the luxI-like genetic contexts. The dendrogram groups contexts by Jaccard distance, with groups defined by an optimal distance cutoff. The outermost boxes indicate the plasmid group, and the inner boxes denote the plasmid category.

SUPPLEMENTARY FIGURE S3 RepABC plasmids and the genetic organization of genes involved in conjugation. Squares at the top of the figure indicates the operons names. Genes involved in quorum sensing are *tra*I (luxI-like) and *tra*R (*lux*R-like). Gene *bis*R is a *lux*R-like solo, and *traR*_2 represents a second copy of *tra*R. Genes in gray denote those not included in the analysis.

SUPPLEMENTARY FIGURE S4 Sub-dendrogram of LuxI-like genetic contexts AE, AG, M, Z, and AA. The colored boxes on the left represent the LuxI-like genomic context (GCO), followed by the corresponding plasmid group number and plasmid category. Adjacent to these, a heatmap illustrates the presence or absence of domains or genes identified within each LuxI-like context. Blue squares show *luxR*-like genes, pink squares show *luxI*-like genes, and yellow squares indicate *dadRXA* gene clusters detected in multiple genomic contexts.

SUPPLEMENTARY FIGURE S5 Sub-dendrogram of LuxI-like genetic contexts R, AF, I, H, and E. The colored boxes on the left represent the LuxI-like genomic context (GCO), followed by the corresponding plasmid group number and plasmid category. Adjacent to these, a heatmap displays the presence or absence of domains or genes identified within each LuxI-like context. Blue squares denote *luxR*-like genes, pink squares denote *luxI*-like genes, magenta squares indicate *rhiABCD* gene clusters detected in multiple genomic contexts, and orange and green squares represent *nod* and *nif* genes, respectively.

SUPPLEMENTARY FIGURE S6 LuxR-like–LuxI-like tanglegram. The mirrored phylogenetic tree illustrates the degree of codivergence between LuxR-like and LuxI-like homologs encoded on chromosomes and plasmids. Lines link LuxR-like LuxI-like cognate partners, and the color on the names and in the lines indicates the degree of codivergence (residuals). The boxes on the left show the genomic context where the QSS resides and, on the right, the plasmid group and category.

SUPPLEMENTARY FIGURE S7 LuxR-LuxI-like pairs residuals distribution. The plot shows the distribution of PACo residuals, with quantiles between the 10th and 99th percentiles used to assess variability and identify potential outliers among LuxI-like and LuxR-like pairs.

SUPPLEMENTARY TABLE S1 Rhizobium reference strains deposited in NCBI. Set of 93 reference strain assembly accessions used to calculate Average Nucleotide Identity (ANI) and to check or correct potential taxonomic discrepancies.

SUPPLEMENTARY TABLE S2 Pfam-A domains of trb and tra operons in the pRetCFN42a plasmid of Rhizobium etli CFN 42. Pfam-A domains used as references to classify potential.
