# Supplementary figures and images for: Diversity and evolution of quorum-sensing systems in Rhizobium
Source: Front Bioinform. 2026 Apr 17;6:1767204. doi: 10.3389/fbinf.2026.1767204 (PMC13133039; doi:10.3389/fbinf.2026.1767204)

Q1=0.38, Q3=1.14, p90=1.35, extreme > Q3+3\*IQR ~ 3.42

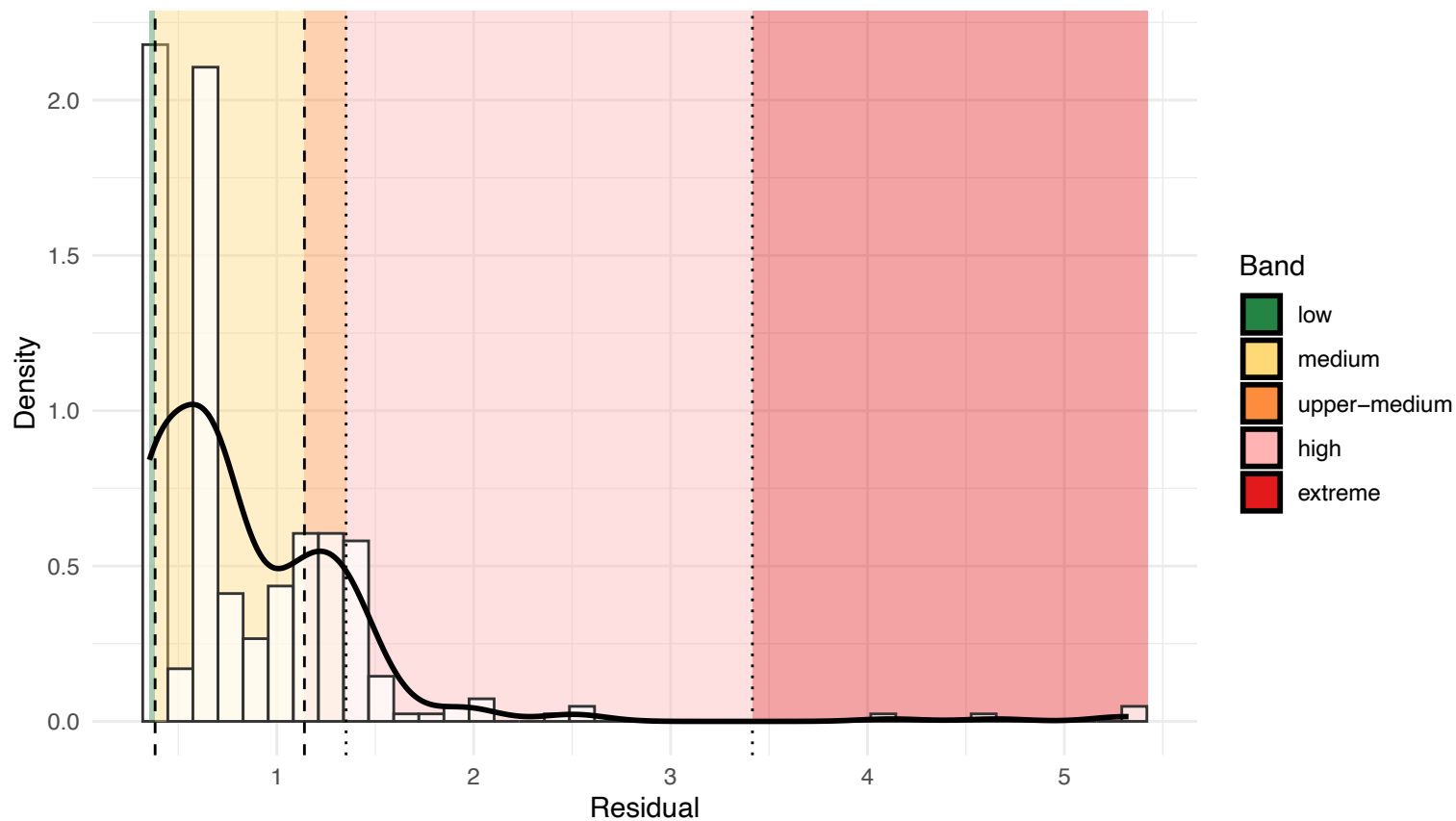

Supplement: Supplementary file 1 [file DataSheet7.pdf]

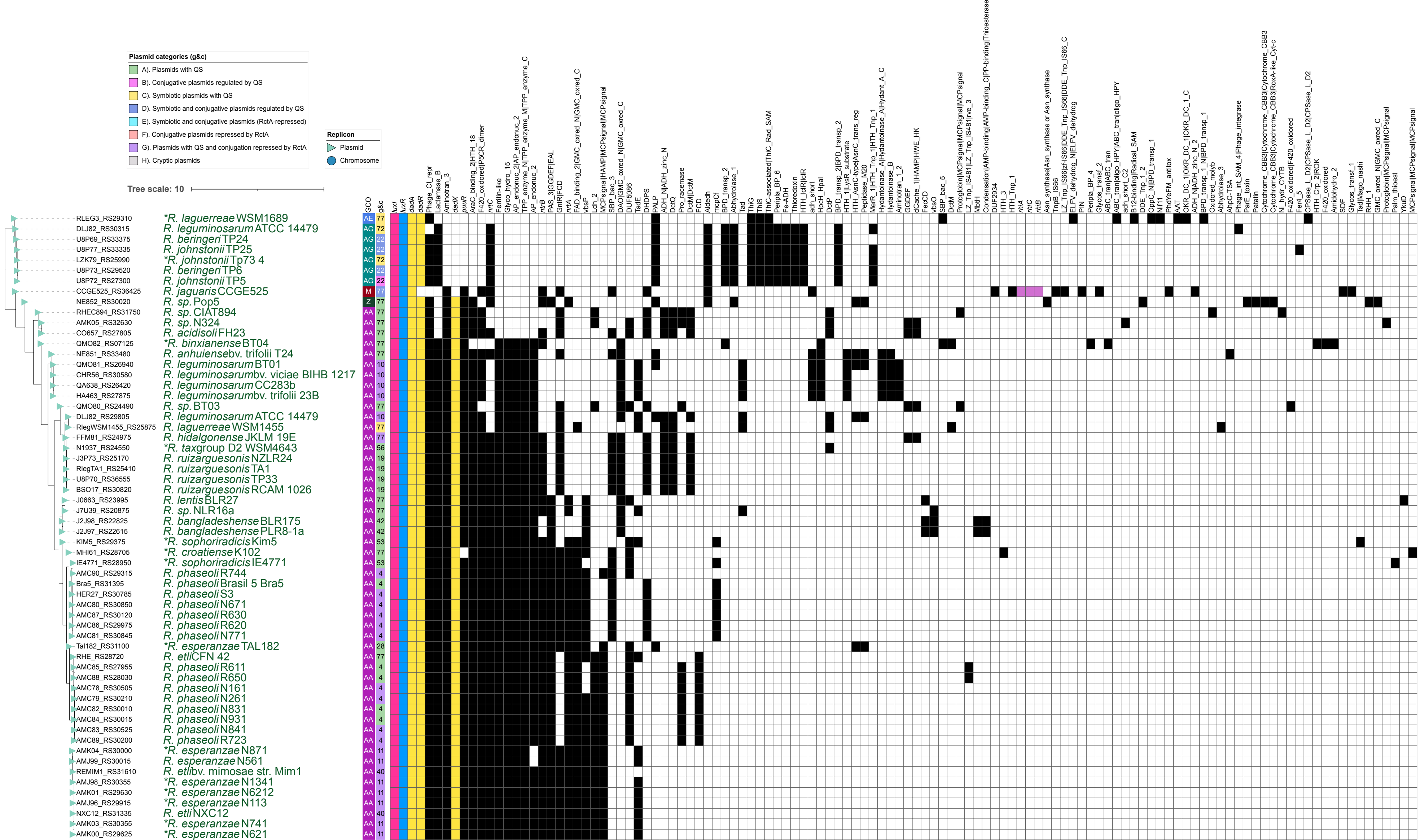

Supplement: Supplementary file 3 [file DataSheet4.pdf]

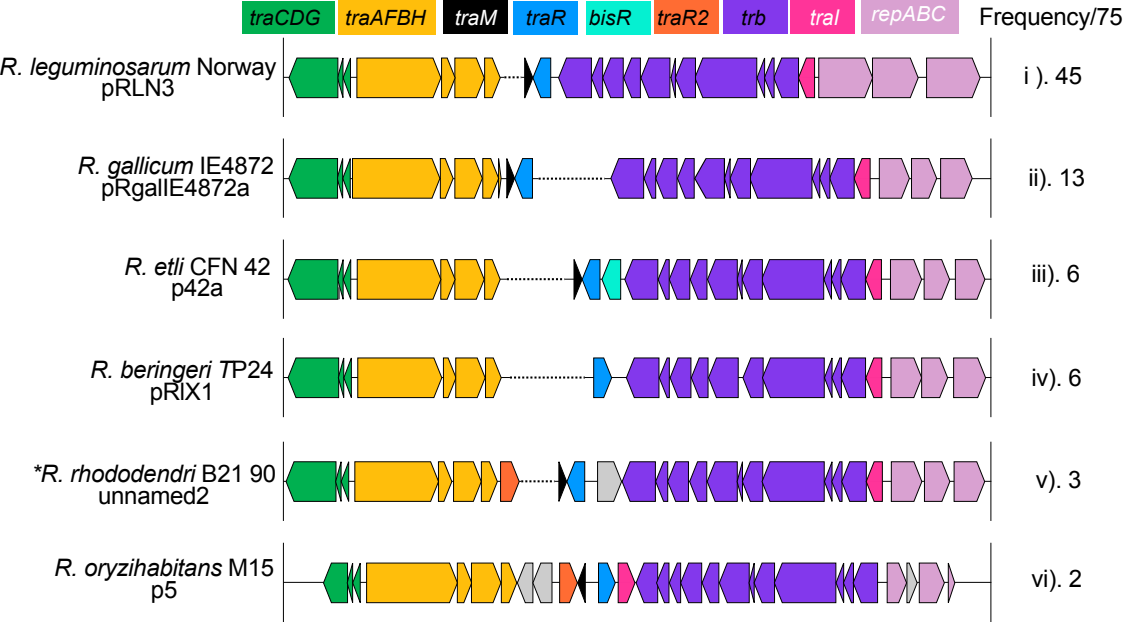

Supplement: Supplementary file 5 [file DataSheet3.pdf]

## Context OUT

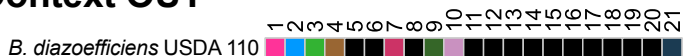

## Context A

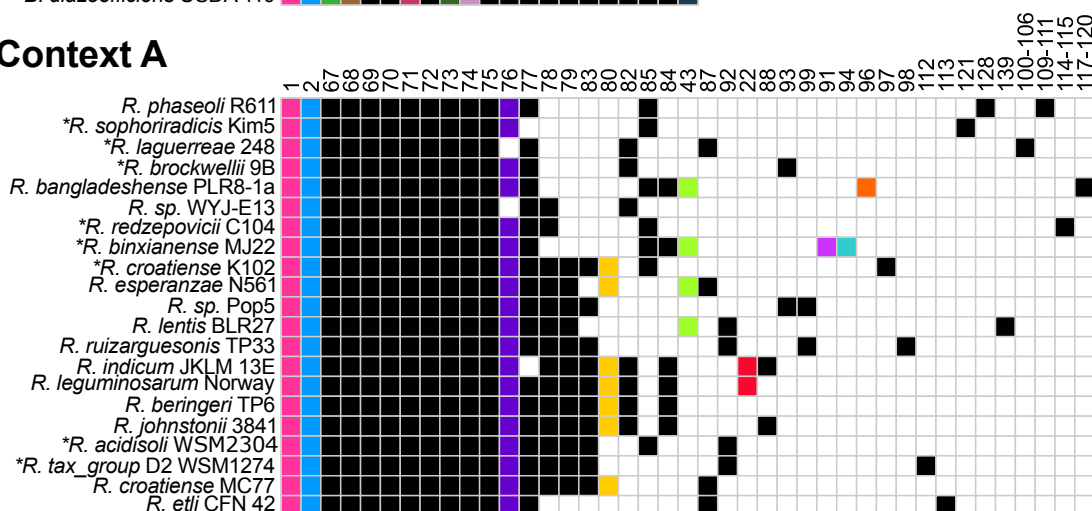

## Context B

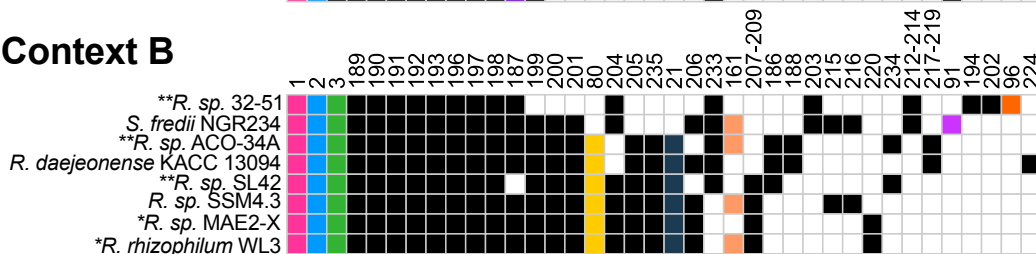

Supplement: Supplementary file 6 [file DataSheet1.pdf]

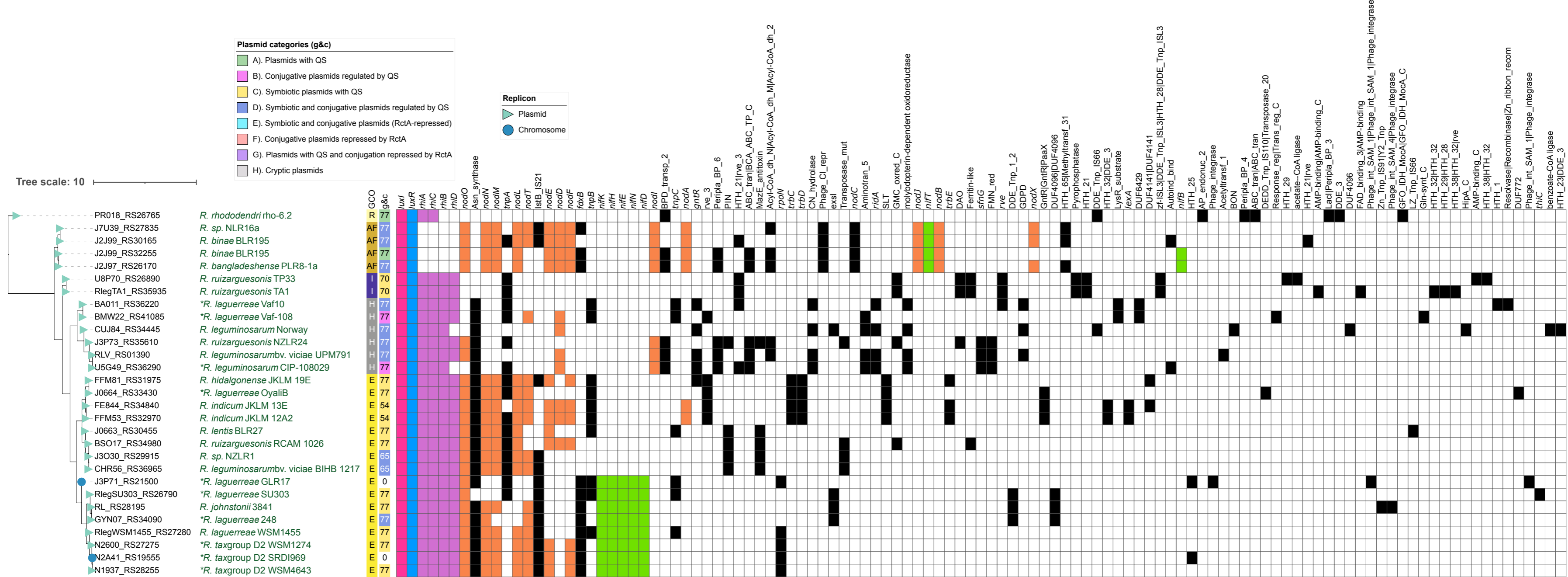

Supplement: Supplementary file 7 [file DataSheet5.pdf]
