# Supplementary material for: Diversity and evolution of quorum-sensing systems in Rhizobium
Source: Front Bioinform. 2026 Apr 17;6:1767204. doi: 10.3389/fbinf.2026.1767204 (PMC13133039; doi:10.3389/fbinf.2026.1767204)
Supplement: Supplementary file 2 [file DataSheet2.pdf]

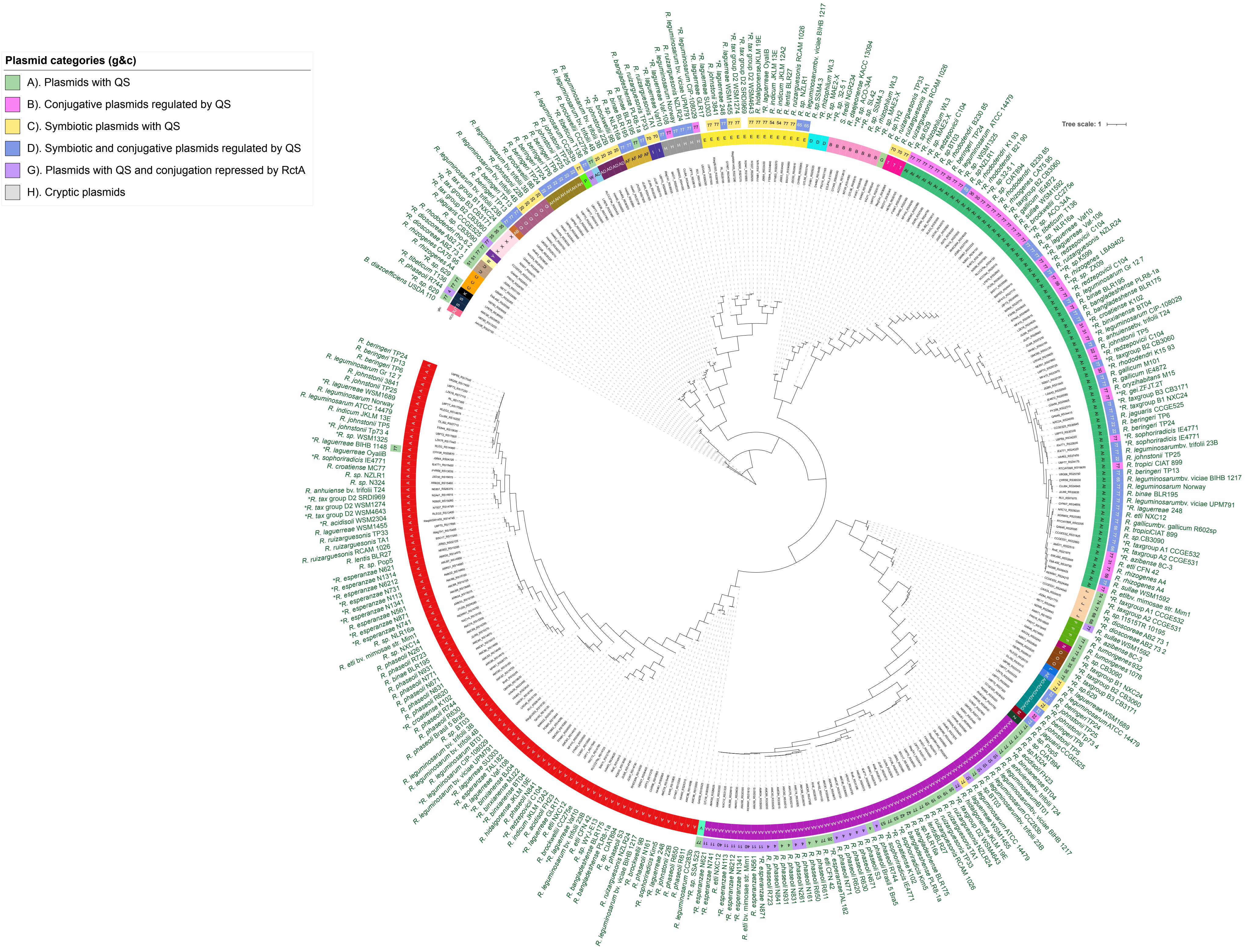
Plasmid categories (g&c)
A). Plasmids with QS
B). Conjugative plasmids regulated by QS
C). Symbiotic plasmids with QS
D). Symbiotic and conjugative plasmids regulated by QS
G). Plasmids with QS and conjugation repressed by RctA
H). Cryptic plasmids
Tree scale: 1
