# Supplementary material for: Diversity and evolution of quorum-sensing systems in Rhizobium
Source: Front Bioinform. 2026 Apr 17;6:1767204. doi: 10.3389/fbinf.2026.1767204 (PMC13133039; doi:10.3389/fbinf.2026.1767204)
Supplement: Supplementary file 4 [file DataSheet6.pdf]

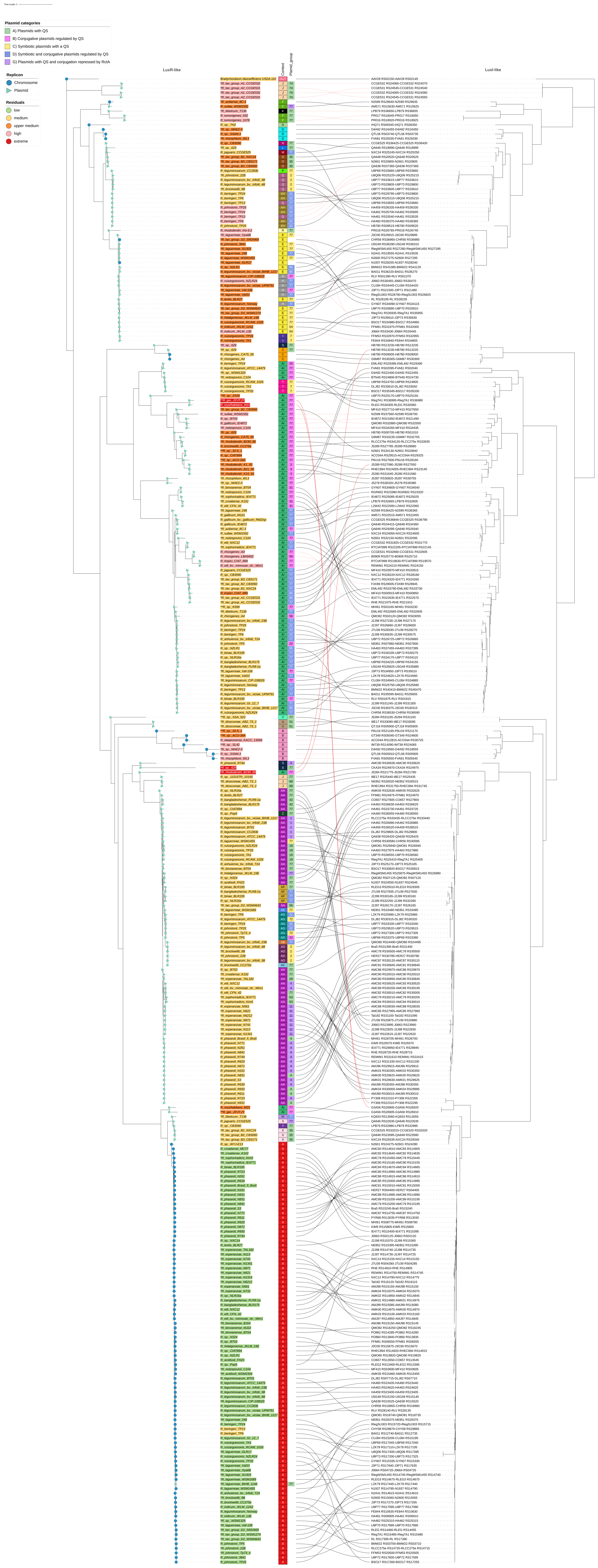

Tree scale: 1
Plasmid categories
A) Plasmids with QS
B) Conjugative plasmids regulated by QS
C) Symbiotic plasmids with a QS
D) Symbiotic and conjugative plasmids regulated by QS
G) Plasmids with QS and conjugation repressed by RctA
Replicon
Chromosome
Plasmid
Residuals
low
medium
upper medium
high
extreme
LuxR-like
LuxI-like
Context
Plasmid_group
